# Using Patient-Pathway Analysis to Inform a Differentiated Program Response to Tuberculosis: The Case of Kenya

**DOI:** 10.1093/infdis/jix381

**Published:** 2017-11-06

**Authors:** Enos Masini, Christy Hanson, Jeremiah Ogoro, Jessie Brown, Faith Ngari, Pia Mingkwan, Julia Makayova, Mike Osberg

**Affiliations:** 1 Ministry of Health National Tuberculosis, Leprosy and Lung Disease Program, Nairobi, Kenya; 2 Macalester College, St. Paul, Minnesota; 3 Bill and Melinda Gates Foundation; 4 Linksbridge, Seattle, Washington

**Keywords:** Tuberculosis, patient-pathway analysis, care seeking, diagnostic, treatment services, public sector, private sector

## Abstract

**Background:**

A recent tuberculosis prevalence survey in Kenya found that the country is home to nearly twice as many patients with tuberculosis as previously estimated. Kenya has prioritized identifying and treating the unnotified or missing cases of tuberculosis. This requires a better understanding of patient care seeking and system weaknesses.

**Methods:**

A patient-pathway analysis (PPA) was completed to assess the alignment between patient care seeking and the availability of tuberculosis diagnostic and treatment services at the national level and for all 47 counties at the subnational level in Kenya.

**Results:**

It was estimated that more than half of patients initiate care in the public sector. Nationally, just under half of patients encountered tuberculosis diagnostic and treatment capacity where they initiated care. Overall, there was distinct variation in diagnostic and treatment availability across counties and facility levels.

**Discussion:**

The PPA results emphasized the need for a differentiated approach to tuberculosis care, by county, and the distinct need for better referral systems. The majority of Kenyans actively sought care; improving diagnostic and treatment capacity in the formal and informal private sector, as well as in the public sector, could help identify the majority of missing cases.

Universal health coverage has been recognized as a catalyst for sustainable development, including poverty reduction and the amelioration of health and well-being. The goal of universal health coverage is to ensure that all people obtain the health services they need without experiencing financial hardship [1]. In 2010, Kenya adopted universal health coverage as the framework for its multisectoral approach to healthcare, with the central value of affordable healthcare as a constitutional right for all Kenyans [2]. The adoption of universal health coverage is particularly significant given Kenya’s economic landscape. Of the estimated 46 million Kenyans, nearly 46% live below the poverty line [3]. Out-of-pocket health spending accounted for approximately 26% of total health expenditures in 2014 [4]. The financial burden of healthcare impoverished some Kenyans and deterred others from seeking the care they needed altogether. Approximately 60% of mortality was due to preventable and treatable diseases in 2015 [5], indicating that if Kenyans had access to appropriate care, most deaths could be prevented. Tuberculosis is one such preventable and treatable disease and is the fourth leading cause of death in Kenya [3].

Goal 3.3 of the Sustainable Development Goals includes the target to end the tuberculosis epidemic by 2030 [6]. In its 2015–2018 National Strategic Plan for Tuberculosis, Leprosy, and Lung Health, Kenya embraced the Sustainable Development Goals objectives and World Health Organization targets to dramatically reduce the impact of tuberculosis in Kenya [3]. Specifically, the country aimed to reduce the incidence of tuberculosis by 5%, reduce mortality due to tuberculosis by 3%, and reduce the proportion of affected families who face catastrophic costs due to tuberculosis by 2018 [3]. These commitments are important in light of a recent tuberculosis prevalence survey that found that Kenya is home to nearly twice as many patients with tuberculosis as previously estimated (Kenya Tuberculosis Prevalence Survey 2015–2016, National Tuberculosis, Leprosy, and Lung Disease Program, Kenya Ministry of Health, unpublished document). These findings emphasize that the challenges to ending tuberculosis are greater than previously assumed.

In 2010, Kenya ratified a new constitution that reorganized the country into 47 semiautonomous counties [2]. Kenya’s national health system mirrors the devolution of power to the county level; the national government takes responsibility for policy development and management of national referral facilities, while the county governments manage county health facilities and healthcare budget allocations. There are nearly 10000 health facilities in Kenya, of which 48% are in the public sector, 38% are in the private sector, and 14% are owned by nongovernmental organizations [2].

The Government of Kenya offers free tuberculosis treatment to all patients through >3000 of its public health facilities [3]. In 2016, the treatment success rate for notified cases was 88% (TIBU patient treatment records, accessed June 2016, National Tuberculosis, Leprosy, and Lung Disease Program, Kenya Ministry of Health, unpublished document). This high figure suggests that public health centers deal effectively with tuberculosis cases once they are recognized and diagnosed. However, approximately one third of all new tuberculosis cases remained unnotified in 2015 [7]. In other words, one third of tuberculosis cases are either never diagnosed or do not receive appropriate treatment. Kenya has prioritized the identification and treatment of these missing cases. The National Tuberculosis, Leprosy, and Lung Disease Program (NTLP) has acknowledged that identifying and curing patients with tuberculosis who are currently missing will require more information with regards to patient care-seeking patterns and health system weaknesses [3].

## METHODS

The patient-pathway analysis (PPA) methods described by Hanson et al elsewhere in this issue was used to assess the alignment between patient care seeking and the availability of tuberculosis diagnostic and treatment services [8]. PPAs were completed at the national level, for urban and rural care seekers, and for each of the 47 counties. The PPAs drew from routinely collected programmatic data and national survey data. The data sources used for the PPAs are described in Table 1. Further background on each data source is provided in the Supplementary Materials.

**Table 1.** Primary Data Sources for the Patient-Pathway Analysis (PPA)

PPA Component and Subcomponent(s)	Data Source
No. of facilities	
Includes formal private and public facilities^a^	2016 Kenya Master Health Facility List [12]
Place of initial care seeking	
Where respondents sought care for tuberculosis and other respiratory illnesses	2013 Kenya Household Expenditure and Utilization Survey [13] (primary source)
Patients who sought care for antenatal HIV test (proxy for tuberculosis)	2014 Demographic and Health Survey [14] (secondary source used for 3 counties not included in KHHEUS)
Tuberculosis diagnostic availability at initial care seeking	
Smear microscopy	2016 Laboratory Records, National Tuberculosis, Leprosy and Lung Disease Program [16]
Xpert machine and Xpert referral	2016 Xpert test records, National Tuberculosis, Leprosy and Lung Disease Program [17]
Radiography	2013 Service Availability and Readiness Assessment Mapping [15]
Tuberculosis treatment availability at initial care seeking	
Any tuberculosis drugs available	2013 Service Availability and Readiness Assessment Mapping [15]

Abbreviations: HIV, human immunodeficiency virus; KHHEUS, Kenya Household Expenditure and Utilization Survey.

^a^No data were available on the no. of informal private facilities.

Each data source used a different naming convention for health facility types. To allow for comparison across data sources, facilities were categorized as public, formal private, or informal private and assigned to one of 5 following levels. Level 1 (L1) facilities offer the most basic care and are usually community based. L1 services include basic triage, provision of health information, and essential prevention and care activities. Services are commonly provided as an extension of facility-based care and are provided by volunteers or paramedical staff with limited formal training. Limited laboratory testing is available in a few cases, and L1 staff may serve as treatment supporters for patients with tuberculosis. Examples include community health worker facilities (public) and traditional healer facilities, mobile clinics, private clinics, and voluntary counseling and testing sites (private). Level 2 (L2) facilities provide the first point of contact with patients and are usually staffed by nurses and public health technicians. L2 facilities provide basic outpatient care. Some diagnostic services and essential medicines may be available. Examples include government dispensaries (public) and pharmacies, nongovernmental organization clinics, and laboratories (private). Level 3 (L3) facilities provide primary healthcare. Nurses, midwives, and private physicians commonly provide L3 services, generally on an outpatient basis. L3 facilities have more-extensive diagnostic and treatment options. Examples are primary health care facilities (public) and nursing homes and mission health centers (private). Level 4 (L4) facilities provide primary healthcare, as well as more-advanced care. L4 facilities commonly provide both outpatient and inpatient care. Examples include government hospitals (public) and hospitals and clinics (private). Level 5 (L5) facilities are teaching and referral hospitals. 

The levels used here are different from those in the other country case studies included in this supplement. Kenya has an existing method of level categorization, which was used for the PPA. Table 2 provides a detailed mapping of the health facilities from each data source to the standard categories described above.

**Table 2.** Health Facility Categorization

Data Source, Health Facility Type	Health Facility Sector	Health Facility Level
2016 Kenya Master Health Facility List		
Not available	Informal private	2
Not available	Informal private	1
Private primary care hospital	Formal private	4
Private secondary care hospital	Formal private	4
Private basic primary health care facility	Formal private	3
Private comprehensive primary health care facility	Formal private	3
Farewell home	Formal private	3
Private administrative office	Formal private	2
Private dispensary and outpatient-only clinic	Formal private	2
Private laboratory	Formal private	2
Radiology clinic	Formal private	1
Private voluntary counseling and testing center	Formal private	1
Public teaching and referral hospital	Public	5
Public hospital	Public	4
Public primary care hospital	Public	4
Public secondary care hospital	Public	4
Public basic primary health care facility	Public	3
Public comprehensive primary health care facility	Public	3
Regional blood transfusion center	Public	3
Public administrative office	Public	2
Public dispensaries and clinic outpatient only	Public	2
Public laboratory	Public	2
Blood bank	Public	1
Public voluntary counseling and testing center	Public	1
2013 Household Health Expenditure and Utilization Survey		
Chemist facility, pharmacy, shop	Informal private	2
Community pharmacy	Informal private	2
Other	Informal private	1
Traditional, religious, cultural healer facilities	Informal private	1
Village health worker (TBA, CHW) facility	Informal private	1
Mission hospital	Formal private	4
Private hospital	Formal private	4
Mission health center	Formal private	3
Nursing, maternity homes	Formal private	3
Company, parastatal clinics	Formal private	2
Mission dispensary	Formal private	2
Nongovernmental organization clinic	Formal private	2
Private clinic	Formal private	2
Government hospital	Public	4
Government health center	Public	3
Government dispensary	Public	2
Not available	Public	1
2014 Demographic and Health Survey		
Shop	Informal private	2
Pharmacy	Informal private	1
Relative, friend	Informal private	1
Traditional practitioner	Informal private	1
Mission hospital, clinic	Formal private	4
Private hospital, clinic	Formal private	4
Not available	Formal private	3
Not available	Formal private	2
Mobile clinic	Formal private	1
Other private sector	Formal private	1
Private doctor	Formal private	1
Government hospital	Public	4
Government health center	Public	3
Government dispensary	Public	2
Community health worker	Public	1
Other public sector	Public	1
2016 TIBU Patient Treatment Records		
Private hospital	Formal private	4
Private health center	Formal private	3
Private maternity home	Formal private	3
Private nursing home	Formal private	3
Private clinic	Formal private	2
Private dispensary	Formal private	2
Foundation	Formal private	2
Private voluntary counseling and testing center	Formal private	1
Private SWOP	Formal private	1
Teaching and referral hospital	Public	5
Public hospital (non–level 5)	Public	4
Public health center	Public	3
Public maternity home	Public	3
Public nursing home	Public	3
Public dispensary	Public	2
Rural health training center	Public	2
Rural health demonstration center	Public	2
Public voluntary counseling and testing center	Public	1
Public SWOP	Public	1

Abbreviation: CHW, community health worker; SWOP, sex workers operation project; TBA, traditional birth attendant.

The 2016 Kenya Master Health Facility List (FML) included 9759 health facilities and was considered comprehensive for public sector and formal private sector facilities in the country. The number of informal private health facilities is unknown. Data on tuberculosis services available at specific facilities captured in the 2013 Kenya Service Availability and Readiness Assessment Mapping (SARAM) and NTLP records were added to the FML, and facilities were matched by numeric identifier.

**Figure 1.**
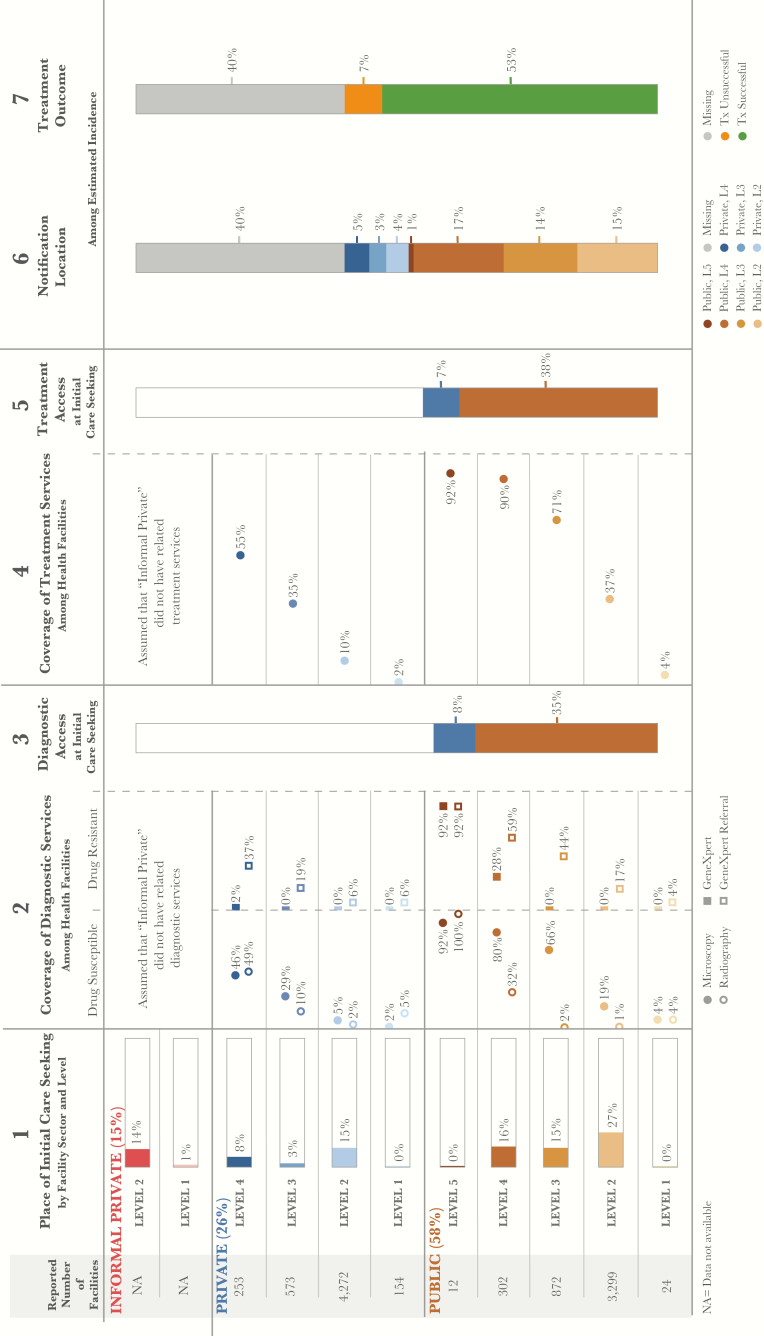
Patient-pathway visual at the national level. The patient pathway describes the care-seeking patterns of patients and how those patients may intersect with tuberculosis services. Column 1 identifies the sectors and levels of the health system, followed by the estimated number of health facilities at each sector and level, according to the 2016 Kenya Master Health Facility List (FML) for Kenya [12]. The percentage next to each sector title is the share of patients who initiate care seeking in this sector. The final part of column 1 shows the percentage of patients seeking care at each sector and level among participants in the 2013 Kenya Household Health Expenditure and Utilization Survey (KHHEUS) who sought care for tuberculosis and/or respiratory illness in the 4 weeks preceding the survey [15]. Three of Kenya’s 47 counties—Garissa, Mandera, and Wajir—were excluded from the KHHEUS and are not reflected in the national pathway. Column 2 shows the percentage of health facilities that have tuberculosis diagnostic tools across each sector and level of the health system. Data on radiography coverage are from 2013 Kenya Service Availability and Readiness Assessment Mapping (SARAM) records [17]. Smear microscopy coverage reflects National Tuberculosis, Leprosy, and Lung Disease Program laboratory records [17]. GeneXpert system and GeneXpert referral coverage reflect national GeneXpert test records (19). Diagnostic tools are separated by tools for diagnosing drug-susceptible tuberculosis and those for diagnosing drug-resistant tuberculosis. Column 3 shows the estimated percentage of patients likely to access a facility with a tuberculosis diagnostic tool on their initial visit to a health care facility, per the care-seeking patterns reflected in the KHHEUS. Data in column 3 were calculated in 2 stages. First, where possible (for 27% of KHHEUS care seekers), the facilities reportedly visited were matched by name to facilities from the FML, and the proportion of care seekers who accessed a facility with any tuberculosis diagnostic tool at initial care-seeking was calculated. Second, for the remainder of care seekers whose place of initial care seeking was not matched by name to the FML, access to diagnosis was estimated on the basis of diagnostic services coverage (columns 2a and 2b) at the health sector and level where they reportedly sought care. By multiplying the proportion of care seekers at each level by the diagnostic coverage at that level (among facilities that were not matched to care seekers in the first stage), the percentage of care seekers who accessed a facility with any diagnostic tool at the initial point of care seeking was estimated. Column 3 reflects the sum of stages 1 and 2—the total proportion of care seekers estimated to have accessed any diagnostic tool at initial care seeking. Columns 3 and 5 separate public and private sectors on the basis of each sector’s contribution to tuberculosis services access at initial care seeking. Column 4 shows the percentage of health facilities that have tuberculosis treatment, reflecting the availability of any tuberculosis drugs when the 2013 SARAM data were collected. Column 5 shows the estimated percentage of patients likely to access tuberculosis treatment at initial care seeking, per KHHEUS care-seeking patterns. Column 5 was calculated in 2 stages in the same manner as in column 3. Column 6 shows which sectors and levels provided case notification, and data were calculated as a share of the overall estimated incidence in 2015. Column 7 shows the treatment outcome of notified cases among the overall estimated incidence for 2015. Data on notification source and treatment outcomes reflect NTLP records [8]. Columns may not add to 100%, owing to rounding. For more details on the data sources used in the pathway, see the Supplementary Materials.

The location of care initiation (column 1, Figure 1) reflects 5521 patients included in Kenya’s 2013 Household Health Expenditure and Utilization Survey (KHHEUS) who sought care for tuberculosis or respiratory illness in the 4 weeks preceding the survey. Three counties—Garissa, Mandera, and Wajir—were not included in the KHHEUS. For these counties, the PPA was based on patient care-seeking data reported in the Demographic and Health Survey (DHS). The DHS did not have a tuberculosis-specific survey question, so human immunodeficiency virus testing location was used as a proxy for tuberculosis care initiation for these 3 counties.

The coverage by diagnostic services at each health facility level (columns 2a and 2b, Figure 1) is determined by a combination of data sources. The numerator was estimated using the NTLP’s records of sites with acid-fast bacilli testing and light-emitting diode microscopy, Xpert, and formalized Xpert referral, as well as the SARAM’s records of radiography services. Facilities from the FML are used as the denominator. A facility was designated as having Xpert referral capacity if the Xpert test records list included at least 1 patient record designating that facility as the facility from which the patient was referred. Most facilities from the NTLP and SARAM records were matched by numeric identifiers to facilities in the FML. However, facilities not found in the FML were excluded from the analysis and not reflected in columns 2a and 2b. These facilities include 149 of 3714 laboratories (4%) providing microscopy data, 127 of 1724 Xpert referral facilities (7%), 1 of 113 Xpert sites (1%), and 1020 of 8041 SARAM facilities (13%).

Access to diagnosis at the point of care initiation (column 3, Figure 1) was calculated in 2 steps. First, where possible, the facilities reportedly visited by patients surveyed in the KHHEUS were matched by name to facilities from the FML. Facilities were matched by name because the KHHEUS did not include numeric facility identifiers. Owing to inconsistencies in facility naming conventions between the KHHEUS and FML, only 27% of patients were matched to the exact facility in the FML where they initiated care. For those 27% of patients, the tuberculosis services available at the facilities visited were captured. Using these data, the proportion of patients who accessed any form of diagnosis at the point of care initiation was calculated.

Second, for the remainder of patients whose location of care initiation was not matched by name to the FML, access to diagnosis was estimated on the basis of the services coverage (columns 2a and 2b, Figure 1) at the health sector and level where the patient reportedly initiated care. By multiplying the proportion of patients initiating care at each level by the availability of any form of diagnosis at that same level (among facilities that were not matched in the first step), the percentage of patients who accessed diagnosis at the initial point of care seeking was estimated. Column 3 in the patient-pathway visual reflects the sum of steps 1 and 2, the total proportion of care seekers estimated to have accessed health facilities with any diagnostic services at the time of initial care seeking.

Column 4 shows the coverage of tuberculosis treatment drugs at each level (Figure 1). The SARAM records provided data on the drugs in stock at each facility at the time of the survey. As noted above, 13% of facilities from the SARAM records were excluded from the analysis because they were not found in the FML. Last, column 5 represents the percentage of patients who visited a facility with tuberculosis treatment available on their first engagement with the health system (Figure 1). The calculations were performed using the same methods from column 3.

Column 6 shows the contribution of each health care sector and level to tuberculosis case notifications as a share of the overall estimated incidence in 2015 (Figure 1). Column 7 shows the treatment outcome of notified cases among the overall estimated incidence for 2015 (Figure 1). Columns 6 and 7 reflect NTLP records.

### Limitations

Several limitations to Kenya’s PPA should be noted. First, 16 counties included survey data that reflected patient care initiation in facilities at levels for which the FML had no listed health facilities, most of which were private formal L1 and L4 facilities. This observation might indicate either that patients initiate care outside their county of residence or that the FML omitted certain private L1 and L4 facilities. The missing facilities would only affect PPA findings if tuberculosis treatment availability existed at a given level that was not captured; the existing methods assume that services are unavailable in settings where care initiation occurs but there are no records of any facilities.

Second, the PPA estimates the likelihood of a patient accessing care on their first visit, using the coverage of diagnostic and treatment services at health facilities, regardless of whether or not a facility has this tool or service. It may be the case that patients who visit a health facility with tuberculosis services does not actually receive that service on their first visit (or at all). Many factors could influence whether a patient receives the service, including the lack of supporting infrastructure, the quality of the service, or the capacity of the health care facility. Thus, the PPA may overestimate the likelihood of a patient receiving care on their first visit if it uses only coverage as a metric.

The PPA may underestimate the availability of treatment in the public sector. An electronic recording and reporting system and corresponding supply chain management system enable the distribution of tuberculosis drugs to sites as patients are notified and initiate treatment. As such, a facility that was not supervising any patients with tuberculosis who were receiving treatment at the time of the SARAM would not be recorded as a treatment facility.

## RESULTS

### Care Initiation

**Figure 2.**
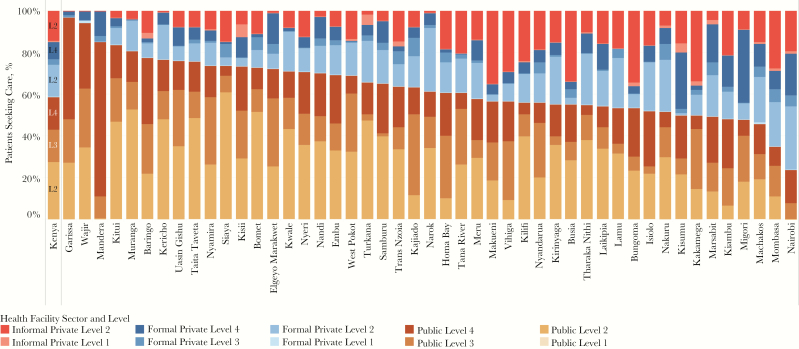
Care-seeking patterns, by county. In addition to the national-level patient-pathway analysis, the patient-pathway analysis was completed at the subnational level for each of the 47 counties in Kenya. The figure depicts the diversity of care-seeking patterns across each of these counties. Care-seeking data for 44 of 47 counties was provided by a question in the 2013 Kenya Household Health Expenditure and Utilization Survey (HHEUS) that asked participants where they sought care for tuberculosis and other respiratory illnesses [13]. The HHEUS was missing data for 3 counties. Data for these counties were provided by the 2014 Demographic and Health Survey (DHS), which asked patients where they sought care for a human immunodeficiency virus test [14]. Each of these care-seeking data sets was categorized to common sector and level categories as described in this articles.

Nationally, over half of patients sought care for tuberculosis symptoms in the public sector, while 26% initiated care in the formal private sector, and an additional 15% went to providers in the informal sector (Figures 1 and 2). Nationally, 58% of patients sought care in lower-level facilities, such as dispensaries (L2) and health centers (L3).

**Figure 3.**
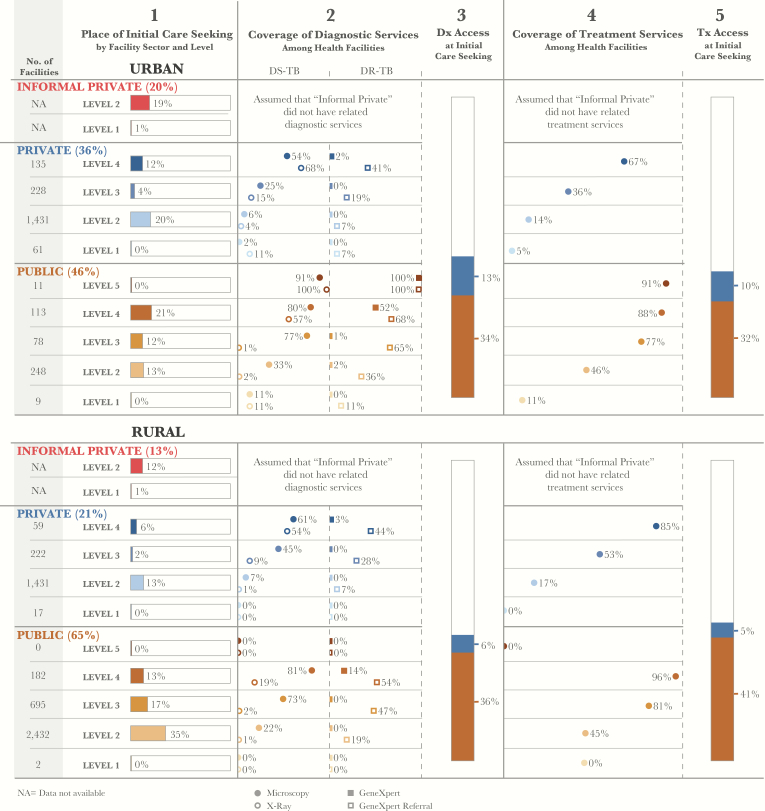
Patient-pathway visual for urban (*A*) and rural (*B*) settings. The urban and rural patient pathways for Kenya use the same underlying methods as the national pathway (Figure 2). The urban or rural designation for care seekers was taken from the 2013 Kenya Household Health Expenditure and Utilization Survey. The urban or rural designation for health facilities was taken from 2013 Kenya Service Availability and Readiness Assessment Mapping (SARAM) records. Data for 25% of facilities from the Kenya Master Health Facility List (2407 of 9761) were either not included in the SARAM records or not given an urban or rural designation in the SARAM records. Thus, the sum of facilities from the urban and rural pathways is a subset of the facilities shown in the national pathway. For example, there are 12 public level 5 facilities in the national pathway, but only 11 of these are reflected in the urban and rural pathways combined. Abbreviation: NA, not available.

Care initiation varied significantly from county to county, across sectors and facility levels (Figure 2). Figure 3 shows considerable urban-rural differences, with considerably higher utilization of the private sector and higher-level public facilities in urban areas. At the county level, care initiation in public hospitals (L4) ranged from 6% of patients in Kilifi to 74% in Mandera. In 8 counties that include large cities, over a third of patients initiated care at formal private facilities. In 4 more-rural counties, including Bungoma, Busia, Kakamega, and Makueni, more than a third of patients initiated care at informal private facilities. The pharmacies and traditional healer facilities that make up the informal sector are not linked to the operations of the NTLP.

### Access to Diagnosis

#### Access to Any Diagnosis Varied Across Counties

**Figure 5.**
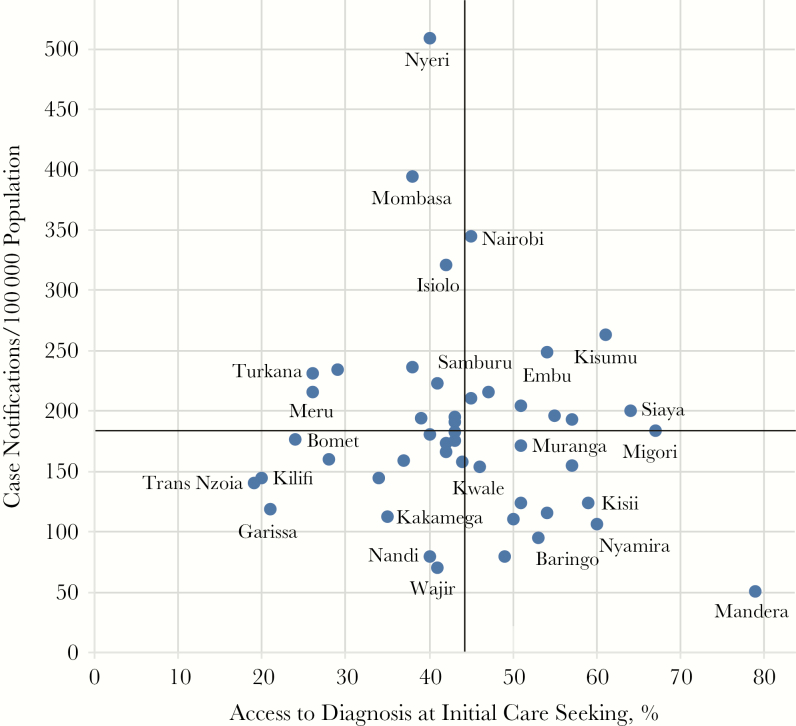
Comparison of rates of case notification to rates of access to diagnosis at initial care seeking. Data on access to diagnosis at the initial point of care seeking for each of the 47 counties are from column 3 of the patient pathway shown in Figure 1. The metric is compared to the 2015 case notification rates for each county. Mean rates of notifications and diagnosis access were 184 notifications/100 population and 44% of patients, respectively.

The availability of any diagnostic services varied between counties and between the private and public sectors. Nationally, 43% of patients encountered at least 1 diagnostic technology at the point of care initiation. In urban areas, 47% of initial visits were at facilities with diagnostic capabilities, but this dropped to 42% in rural areas (Figure 3). At the county level, access to any diagnostic service at the initial point of care seeking ranged from a low of 19% of patients in Trans Nzoia to a high of 79% in Mandera (Figure 5). In 8 counties, <30% of patients had access to any diagnostic technology, while in 35 counties (74%), between 31% and 60% of patients were able to access a diagnostic technology.

#### Microscopy Availability and Patients’ Care Initiation Patterns

There was a relatively high availability of microscopy services in public health centers (L3; 66%) and public hospitals (L4; 80%). However, only 40% of patients who initiated care at a public or formal private provider accessed a facility with microscopy services. Microscopy is considered a pragmatic tool for diagnosis in remote areas and was available in greater proportions of both public and private facilities in rural areas, compared with urban areas.

However, when considering the alignment of microscopy availability to the location of care initiation, some important gaps remained. In rural areas, 35% of patients initiated care in dispensaries (L2), of which only 22% had microscopy available, suggesting thousands of missed opportunities for early diagnosis of tuberculosis. In comparison, >20% of patients in urban areas initiated care in public hospitals (L4), of which 80% had microscopy. In just over half of the counties (55%), all public hospitals had microscopy, suggesting geographic disparities related to infrastructure.

#### Radiography Value and Availability in Public and Private Sectors

The greatest urban-rural disparities in access to diagnosis involved the type of technology available. The recent prevalence survey found radiography to be a highly sensitive test and useful as a tuberculosis screening tool. However, as demonstrated in Figure 1, radiography access was only available to 8% of all patients at the time of care initiation. Radiography was available in hospitals in the public sector and in a limited number of hospitals and clinics in the private sector. Among hospitals in rural areas, 19% of public hospitals (L4; which served 13% of patients) and around half of private hospitals (which served only 6% of patients) had radiography. In half of the counties, <50% of public hospitals (L4) had radiography.

#### Xpert and Microscopy Referral and Coverage

**Figure 4.**
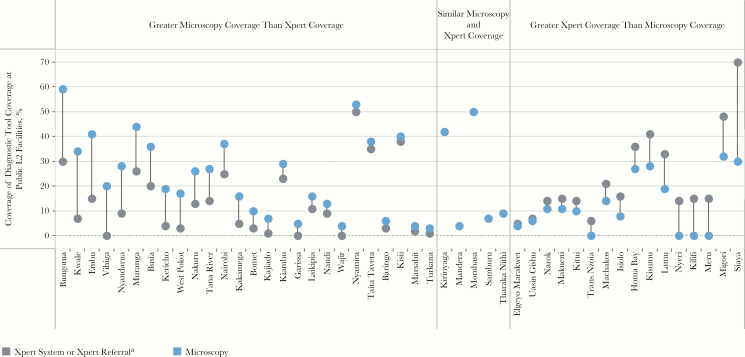
Comparison of smear microscopy and GeneXpert diagnostic coverage in level 2 (L2) public sector facilities. L2 public sector facilities (mostly dispensaries) are the most common location where patients initiate their care-seeking journey. Nationally, only 19% of these facilities have microscopy available, and 17% are connected to other facilities for referral to undergo Xpert testing. This visual compares the diverse levels of microscopy coverage and Xpert system or referral coverage among these facilities at the county level. ^a^For each county, data reflect the variable (systems or referrals) with the greatest level of coverage.

The prevalence survey confirmed the superior role of Xpert in providing bacteriological confirmation of tuberculosis, compared with microscopy. Xpert is also an essential tool for drug-resistance screening. Like radiography, Xpert systems were only available in hospitals (L4), which were visited by 34% of urban patients and 19% of rural patients. To extend the reach of the available Xpert systems, the NTLP has initiated an Xpert referral network to formally link sites that do not have Xpert to a designated Xpert-equipped facility. Nationally, 17% of public dispensaries (L2) referred patients to Xpert sites, as did 44% of health centers (L3) and 59% of hospitals (L4). Multiplying the proportion of patients at each level by the microscopy and Xpert referral coverage at those levels, it was found that 33% of patients could access microscopy at the point of care initiation, compared with 26% of patients who could access Xpert via a referral at the point of care initiation. Figure 4 shows that in counties like Siaya, Xpert referral coverage is more extensive than microscopy coverage, at least in public dispensaries where the majority (61%) of patients in that county seek care.

#### Relationship Between Access to Diagnosis at Point of Care Initiation and Case Notification

Early access to a diagnostic technology should increase case notification rates. However, this relationship was not confirmed. In Figure 5, case notification rates were compared to the percentage of patients who initiated care in facilities with any diagnostic services, including referral. The counties in the upper right and lower left quadrants suggest improved case notification based on early access to diagnostic services. However, the counties in the lower right quadrant have relatively good diagnostic service coverage but below average case notification rates.

### Access to Treatment

Nationally, 45% of patients initiated care in facilities that had the capacity to support their tuberculosis treatment. Tuberculosis treatment was more likely to be available in higher-level facilities, although fewer people initiated care in those facilities. In the public sector, treatment was available for 92%, 90%, 72%, and 37% of patients in hospitals (L5 and L4), health centers (L3), and dispensaries (L2), respectively. Within formal private facilities, treatment was available for 55%, 35%, and 10% of patients in hospitals (L4), health centers (L3), and clinics (L2), respectively.

As with diagnostic access, access to treatment services varied across counties and between urban and rural populations. People living in rural areas were slightly more likely to access treatment where they initiated care than those in urban areas. This was due to greater treatment availability across sectors and levels in rural areas, as well as to higher rates of care initiation in the public sector for rural communities, where treatment availability was comparatively higher than in the private sector. At the county level, the percentage of patients who could access treatment where they initiated care ranged from a high of 66% in Siaya to a low of 24% in Wajir. In 27 counties (57%), treatment was available where 40%–55% of patients initiated care. In an additional 4 counties (8%), >60% of patients initiated care where treatment was accessible.

In more than a third of counties, <90% of public hospitals (L4) provided treatment. For the 27% of patients who initiated care in public dispensaries (L2), only 37% could be treated at that level. Similar misalignment was noted at the county level. For example, only 50% and 46% of public hospitals (L4) in Samburu and Nairobi, respectively, provided treatment, despite these facilities being the second most utilized facilities in both counties. In 13 counties, >10% of people sought care in private clinics, none of which offered treatment services. The pathways revealed important misalignment that could be remedied through a targeted expansion of treatment services to better meet patients where they initiate care.

## DISCUSSION

The KHHEUS found that, of individuals who reported illness during the 4 weeks preceding the survey, >87% had consulted a healthcare service provider for their symptoms. Given the backdrop of active care seeking in Kenya, the PPA findings present an opportunity to better align services to best meet patients where they are within the health system and to thereby reduce the proportion of patients with tuberculosis who do not receive a diagnosis or quality treatment. The PPA generated the following key findings for Kenya.

### County-Level Differences Warrant Differentiated Approaches

The PPAs reflect important urban-rural and intracounty differences in care-seeking patterns and access to tuberculosis services. While the 2015–2018 National Strategic Plan for Tuberculosis, Leprosy, and Lung Health introduced differentiated approaches based on varying epidemiologic contexts throughout the country, the next national strategic plan will capitalize on the evidence emerging from the PPAs to inform differentiated approaches and prioritization of investments that respond directly to patient preferences and needs [3]. Figure 5, for example, will prompt county-level exploration of how gaps in the patient care continuum may affect case notifications. Some possible explanations for low case notification in counties with well-aligned access to diagnostic technologies include the following: (1) providers have a low index of suspicion or refer an insufficient number of patients for diagnostic testing, (2) a high number of patients who receive a tuberculosis diagnosis in the private sector are not notified, (3) a high proportion of people living with tuberculosis never seek care, (4) the prevalence of tuberculosis is lower than average, (5) estimates of access to diagnostic testing are inflated if facilities without testing see larger volumes of patients per facility, and (6) facilities with diagnostic capacity have supply stock-outs or dysfunctional equipment. In counties such as Mombasa, where case notification rates were high but diagnostic infrastructure was lacking, increasing access to diagnostic technologies may yield even more cases, given the underlying high prevalence of tuberculosis in this crowded, urban setting (Kenya Tuberculosis Prevalence Survey 2015–2016, National Tuberculosis, Leprosy, and Lung Disease Program, Kenya Ministry of Health, unpublished document).

Some of the county-level variances may reflect differences in the underlying health system’s capacity. For example, Bungoma and Busia are neighboring counties with similar epidemiologic and socioeconomic profiles, as well as similar rates of access to diagnosis (Figure 5). However, the tuberculosis case notification rate in Busia is considerably higher than it is in Bungoma [2]. General outpatient facility utilization per capita in Bungoma is 0.27 as compared to 0.94 in Busia, which may suggest underlying weaknesses in the health system or health knowledge within the population that are not captured directly by the PPA [9].

### PPA May Inform Multidrug-Resistant Tuberculosis Diagnosis and Treatment Scale-up

Kenya is scaling up diagnosis and care for multidrug-resistant tuberculosis. In 2015, just over a quarter of the 1400 estimated patients with multidrug-resistant tuberculosis (among notified patients with pulmonary tuberculosis) initiated treatment [7]. The PPA demonstrated the importance of continuing to expand the referral network that formally links all public facilities to an Xpert facility, to operationalize the national policy promoting Xpert as the initial diagnostic test for all patients presumed to have tuberculosis. In addition, Kenya has fully decentralized the care of patients with drug-resistant tuberculosis [10]. Wherever a patient with multidrug-resistant tuberculosis is identified or wants to be treated, the capacity of the health staff is built, and second-line drugs are made available. The PPAs suggest where counties can focus their capacity building efforts, in anticipation of and alignment with care-seeking patterns.

### Growing Private Sector Calls for Attention

Kenya has been implementing various public-private mix initiatives over the past 15 years. However, there has been limited engagement beyond hospitals and clinicians in urban areas [11]. While 42% of patients initiated care in the private sector, only 20% of tuberculosis notifications came from the private sector in 2015 [3]. The PPA highlights a key gap in the country’s private sector engagement. The informal sector, including pharmacies, is the initial point of care for 15% of patients presumed to have tuberculosis. Several nongovernmental organization–led initiatives to engage this cadre have been successful but not sustained. In response, the country has reallocated funding from its Global Fund grant to intensify public-private partnerships, including those involving pharmacies, in 5 counties. This funding will enable, for example, the provision of Xpert cartridges for free testing of patients referred from the private sector to Xpert sites. The PPA results will be used to refine the targeting of this effort to facilities with high utilization.

### Efficient Specimen Referral Can Be Built on an Integrated Platform

The PPA has illustrated the misalignment between key technologies—namely Xpert and radiography—and care seeking. Specimen referral systems, rather than patient referral, are essential to close these gaps and accelerate access to diagnosis. Existing specimen transport systems for human immunodeficiency virus load testing have been developed in nearly every county. Other disease control and public health programs are similarly seeking to optimize the use of sophisticated technology. As such, the NTLP will join other efforts to build integrated specimen transport networks across the country.

### Reconsideration of What Diagnostic Coverage Looks Like

Where the prevalence survey highlighted the sensitivity of radiography as a screening tool for tuberculosis, the PPA showed the limited access to radiography where patients with tuberculosis seek care. Last year, the Kenya Ministry of Health procured and distributed 100 additional radiography devices to counties. This is unlikely to dramatically close the gap, as patients with tuberculosis must still travel to higher-level facilities to access these devices. Going forward, the NTLP will consider how to best deploy digital radiography machines, possibly on a mobile basis, to cover dispensaries and health centers where access to fixed radiography machines is constrained by distance and terrain.

## Supplementary Data

Supplementary materials are available at *The Journal of Infectious Diseases* online. Consisting of data provided by the authors to benefit the reader, the posted materials are not copyedited and are the sole responsibility of the authors, so questions or comments should be addressed to the corresponding author.

## Supplementary Material

Supplementary Appendix

## Notes

***Supplement sponsorship.*** This work is part of a supplement sponsored by the Bill & Melinda Gates Foundation.

***Potential conflicts of interest.*** All authors: No reported conflicts of interest. All authors have submitted the ICMJE Form for Disclosure of Potential Conflicts of Interest. Conflicts that the editors consider relevant to the content of the manuscript have been disclosed.
