# Supplementary material for: Using Patient-Pathway Analysis to Inform a Differentiated Program Response to Tuberculosis: The Case of Kenya
Source: J Infect Dis. 2017 Nov 6;216(Suppl 7):S714–23. doi: 10.1093/infdis/jix381 (PMC5854007; doi:10.1093/infdis/jix381)
Supplement: Supplementary Appendix [file jix381_suppl_supplementary_appendix.docx]

| **Country: Kenya** | | | | | |
| --- | --- | --- | --- | --- | --- |
| **Data Source** | **Survey Type** | **Survey Question** | **Reported Metric** | **Sample Size** | **PPA Step** |
| 2016 Facility Master List (FML)  Includes formal private and public facilities;  No informal private facilities | Kenya Master Health Facility List (KMHFL) is an application with all health facilities and community units in Kenya.  <http://kmhfl.health.go.ke/> | For each health facility, data incorporated in the PPA includes:   - Facility name - Facility type - Owner (Ministry of Health, private company, etc.) - County - Numeric ID | Number of health facilities in each sector and level (assigned according to facility type and owner) | n=9,761  Number of listings on the Kenya Master Health Facility List  (accessed January 2017) | Number of facilities |
| Primary source:  2013 Household Health Expenditure and Utilization Survey (HHEUS) | The HHEUS surveyed n=33,675 households, and designed to be representative at the national and county levels, and for rural and urban areas. It uses the National Sample Survey and Evaluation Programme (NASSEP) V sample design, which did not include Mandera, Wajir, and Garissa counties and are thus not included in the sample. | The interviewer asked participants (heads of households) whether members of their household were ill over the last 4 weeks. Nationally, 19% of household members were reportedly ill. Participants indicated the type of illness for each household member who was ill. Individuals for whom their head of household reported “diseases of respiratory including  pneumonia” (n= 5,334,) or “TB” (n= 187) were included in the analysis. For those who reportedly sought care, the interviewer captured:   - Place of care seeking for the last 4 visits to a healthcare provider (name of facility and facility type) - Urban or rural status of the individual - County of residence of the individual   Of the total 5,521 individuals who sought care for respiratory illness or TB, 31% had more than one visit. Only the first visit was included in the patient pathway analysis.  Page 20 of the HHEUS Report notes that illnesses “are self-reported and in many cases are not based on diagnoses by medical personnel. Although the proportions generated through self-reported illness may not reflect similar proportions reported through the routine health information systems, they are  nevertheless informative because they show why individuals may have had contact with health  providers.” | Proportion of care seeking at each sector and level of the healthcare system for people with symptoms of respiratory illness or TB (assigned according to facility type) | n=33,675 households  surveyed  Nationally  n (unweighted) =5,521 individual household members reportedly sought care for respiratory illness or TB, excluding Garissa, Mandera & Wajir counties | Step 1 –  Place of initial care seeking by people with possible TB |
| Secondary source used for 3 counties not included in HHEUS:  2014 Demographic and Health Survey (DHS)  Patients who sought care for antenatal HIV test (proxy for TB) | n= 40,300 households from 1,612 clusters spread across the country, with 995 clusters in rural areas and 617 in urban areas. The 2014 KDHS was designed to produce representative estimates for most of the survey indicators at the national level, for urban and rural areas separately, at the regional (former provincial)  level, and for selected indicators at the county level. | 13.7.2 HIV Counselling and Testing During Pregnancy  “Sixty-eight percent of women who gave birth in the two years before the survey received HIV counselling during antenatal care (ANC). Almost 7 in 10 women (69 percent) were tested for HIV during antenatal care and received the test results and post-test counselling, while 23 percent received results but did not receive post-test counselling. Less than 1 percent of women were tested for HIV during an ANC visit but did not receive the test results.” (p. 239) The type of facility where women received an antenatal HIV test was captured for each woman that received one. | Proportion of care seeking at each sector and level of the healthcare system among pregnant women counselled and tested for HIV | Number of women who sought care for antenatal HIV test:  Garissa n (unweighted) = 185  Mandera n (unweighted) = 97  Wajir n (unweighted) = 162 | Step 1 –  Place of initial care seeking by people with possible TB |
| 2016 Lab Records from National Tuberculosis and Leprosy Programme (NTLP) | 2016 data on all TB labs nation-wide. | For each facility:   - Indicates whether each facility has smear microscopy available (yes/no) - Name of facility - Numeric facility ID | Proportion of facilities with microscopy available at each sector and level of the healthcare system | N = 3,422 labs with data on microscopy availability | Step 2a – Diagnostic availability at initial care seeking (drug sensitive) |
| 2016 Xpert Test Records from NTLP | Data on all Xpert tests nation-wide for the first half of 2016. | Indicates the following for each Xpert test:   - Facility where the test took place (by name and numeric ID) - Facility that referred the patient (by name and numeric ID) | Proportion of facilities with GeneXpert and GeneXpert referral available at each sector and level of the healthcare system | N = 65,534 GeneXpert tests | Step 2b – Diagnostic availability at initial care seeking (drug resistant) |
| 2013 Service Availability and Readiness Assessment Mapping (SARAM) | National Census involving all formal health facilities (public, and private) and management levels in Kenya that was carried out during the period April – May 2013. A standard set of data was collected from each facility and management unit based on a pre-designed questionnaire that collected three sets of information: Basic Information, Service Availability, Service Readiness | Indicates the following for each health facility:   - Facility name - Numeric facility ID - Whether the facility has X-ray (yes/no) - Whether the facility had the following TB drugs available at the time of the survey (yes/no for each): rifampicin, isoniazid, isoniazid + rifampicin (2 fixed dose combination), isoniazid + rifampicin + ethambutol (RHE; 3 fixed dose combination), isoniazid + rifampicin + pyrazinamide + ethambutol (4 fixed dose combination), streptomycin 1 gm injection (for retreatment) - Urban or rural designation | Proportion of facilities with X-ray at each sector and level of the healthcare system  Proportion of facilities with any TB drug available at each sector and level of the healthcare system | n=7995  Number of health facilities nation-wide | Step 2a – Diagnostic availability at initial care seeking (drug sensitive)  Step 4 – Treatment availability at initial care seeking |
| 2016 Prevalence survey infographic | Nationally representative survey of TB prevalence with 63,000 people screened at the community level in 45 of Kenya’s 47 counties | N/A | Estimated TB incidence: 138,105  Notified cases:  82,000  Percentage of missing cases: 40% | N = 63,000 individuals screened; Exact number of positive TB cases not reported | Step 6 – Notification source  +  Step 7  Treatment outcome |
| TIBU TB patient treatment records (NTLP) | All patients notified and treated for TB in early-mid 2016 | Patients with the following outcomes were designated “treatment successful”:   - Treatment completed (54%)   Patients with the following outcomes were designated “treatment unsuccessful”   - Failed treatment (<1%) - Defaulted from treatment (4%) - Loss to follow up (3%)   Patients with the following outcomes were removed from the calculation:   - Blank/missing data (<1%) - Treatment not complete (33%) - Other (5%) | Contribution of each sector and level of the healthcare system to case notification;  Treatment outcomes among notified cases (successful and unsuccessful) | N = 76,531 patients notified and treated for TB | Step 6 – Notification source  +  Step 7 –  Treatment outcome |
